# Supplementary material for: The intervening domain is required for DNA-binding and functional identity of plant MADS transcription factors
Source: Nat Commun. 2021 Aug 6;12:4760. doi: 10.1038/s41467-021-24978-w (PMC8346517; doi:10.1038/s41467-021-24978-w)
Supplement: Supplementary file 1 — Supplementary Information [file 41467_2021_24978_MOESM1_ESM.pptx]

## Slide 1
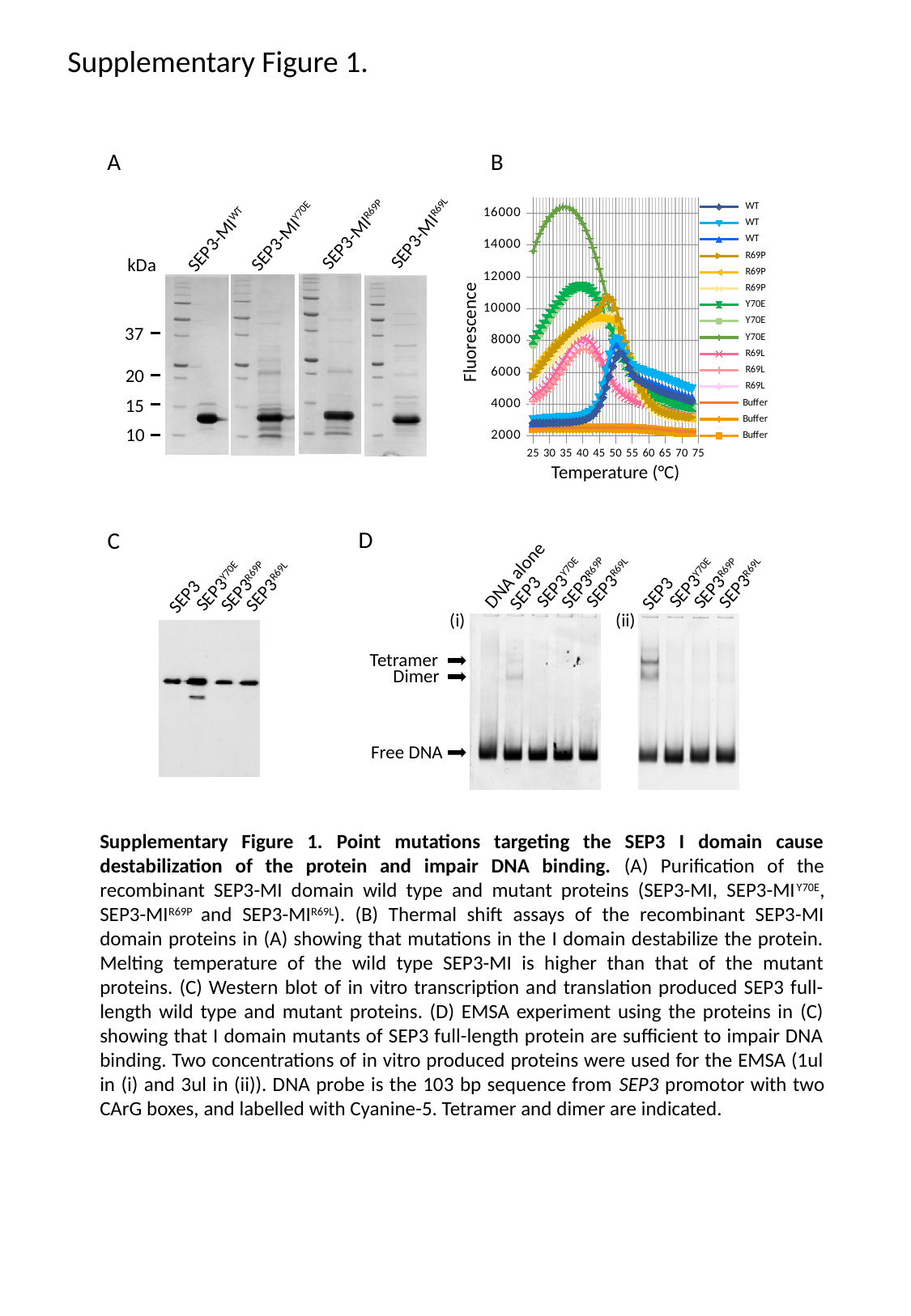

Supplementary Figure 1.
A
B
### Chart
| Category | WT | WT | WT | R69P | R69P | R69P | Y70E | Y70E | Y70E | R69L | R69L | R69L | Buffer | Buffer | Buffer |
|---|---|---|---|---|---|---|---|---|---|---|---|---|---|---|---|SEP3-MIR69L
SEP3-MIR69P
SEP3-MIY70E
SEP3-MIWT
kDa
Fluorescence
37
20
15
10
Temperature (°C)
D
C
DNA alone
SEP3Y70E
SEP3R69L
SEP3R69P
SEP3Y70E
SEP3R69L
SEP3R69P
SEP3Y70E
SEP3R69L
SEP3R69P
SEP3
SEP3
SEP3
(i)
(ii)
Tetramer
Dimer
Free DNA
Supplementary Figure 1. Point mutations targeting the SEP3 I domain cause destabilization of the protein and impair DNA binding. (A) Purification of the recombinant SEP3-MI domain wild type and mutant proteins (SEP3-MI, SEP3-MIY70E, SEP3-MIR69P and SEP3-MIR69L). (B) Thermal shift assays of the recombinant SEP3-MI domain proteins in (A) showing that mutations in the I domain destabilize the protein. Melting temperature of the wild type SEP3-MI is higher than that of the mutant proteins. (C) Western blot of in vitro transcription and translation produced SEP3 full-length wild type and mutant proteins. (D) EMSA experiment using the proteins in (C) showing that I domain mutants of SEP3 full-length protein are sufficient to impair DNA binding. Two concentrations of in vitro produced proteins were used for the EMSA (1ul in (i) and 3ul in (ii)). DNA probe is the 103 bp sequence from SEP3 promotor with two CArG boxes, and labelled with Cyanine-5. Tetramer and dimer are indicated.

## Slide 2
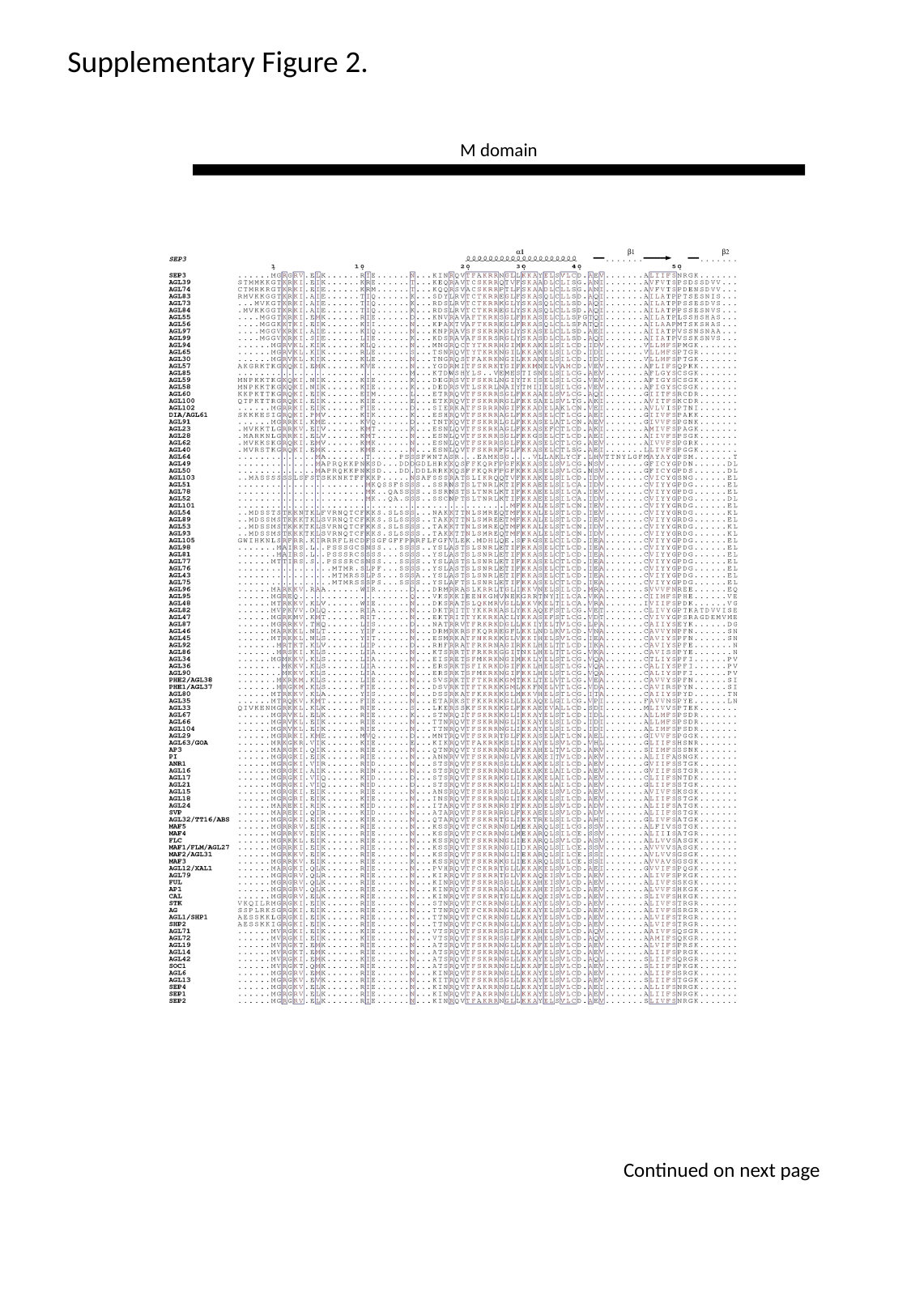

Supplementary Figure 2.
M domain
Continued on next page

## Slide 3
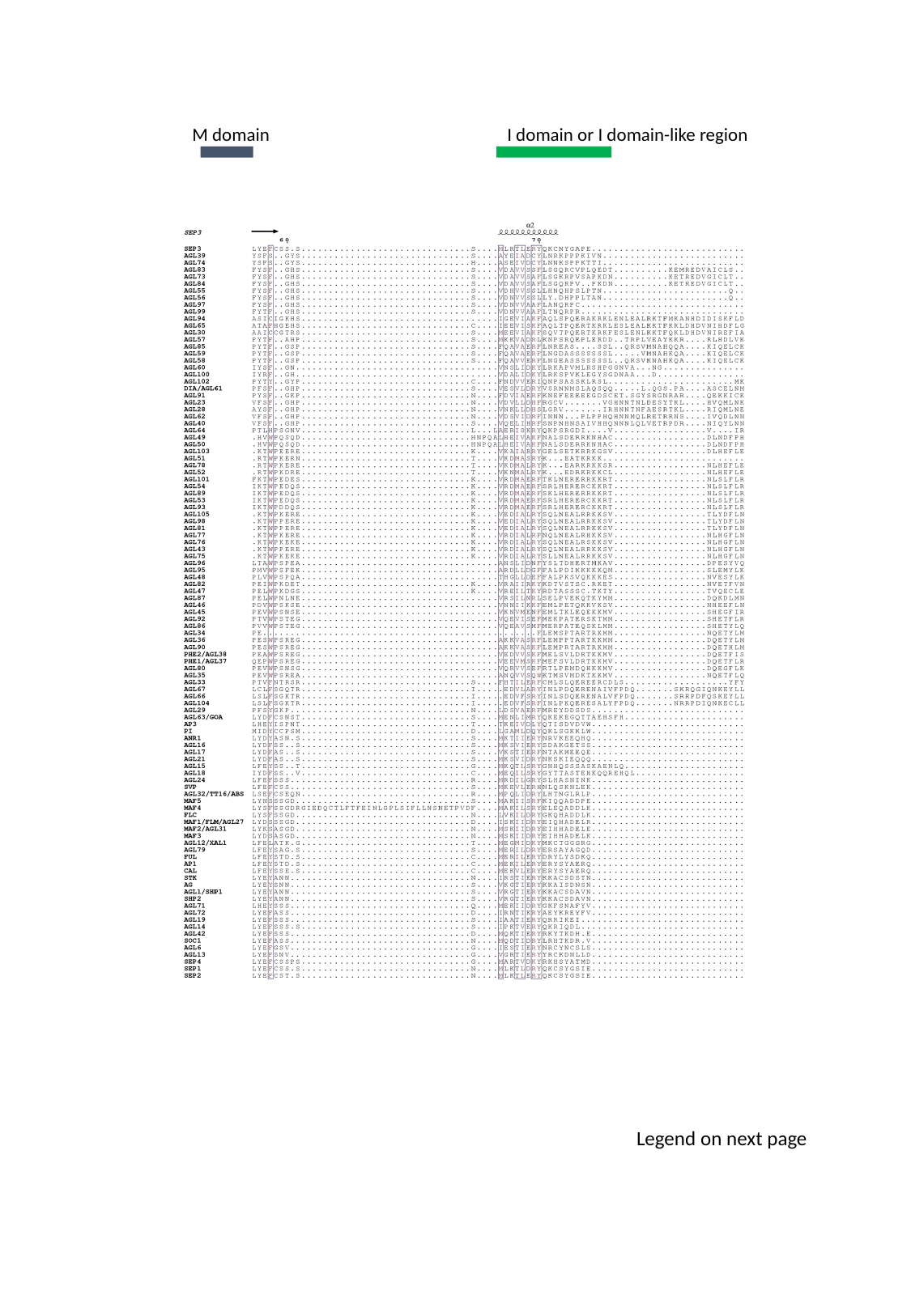

M domain
I domain or I domain-like region
Legend on next page

## Slide 4
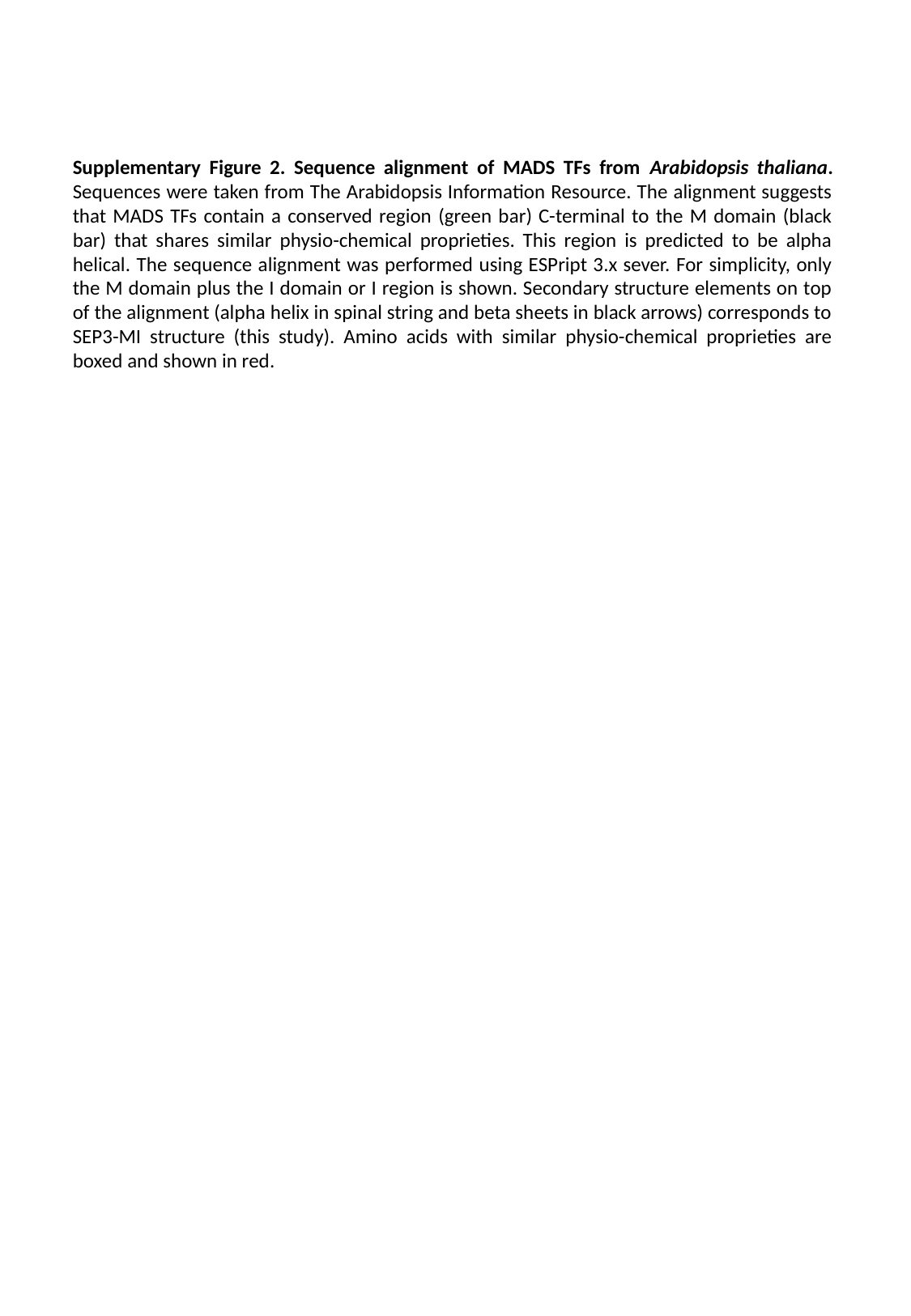

Supplementary Figure 2. Sequence alignment of MADS TFs from Arabidopsis thaliana. Sequences were taken from The Arabidopsis Information Resource. The alignment suggests that MADS TFs contain a conserved region (green bar) C-terminal to the M domain (black bar) that shares similar physio-chemical proprieties. This region is predicted to be alpha helical. The sequence alignment was performed using ESPript 3.x sever. For simplicity, only the M domain plus the I domain or I region is shown. Secondary structure elements on top of the alignment (alpha helix in spinal string and beta sheets in black arrows) corresponds to SEP3-MI structure (this study). Amino acids with similar physio-chemical proprieties are boxed and shown in red.

## Slide 5
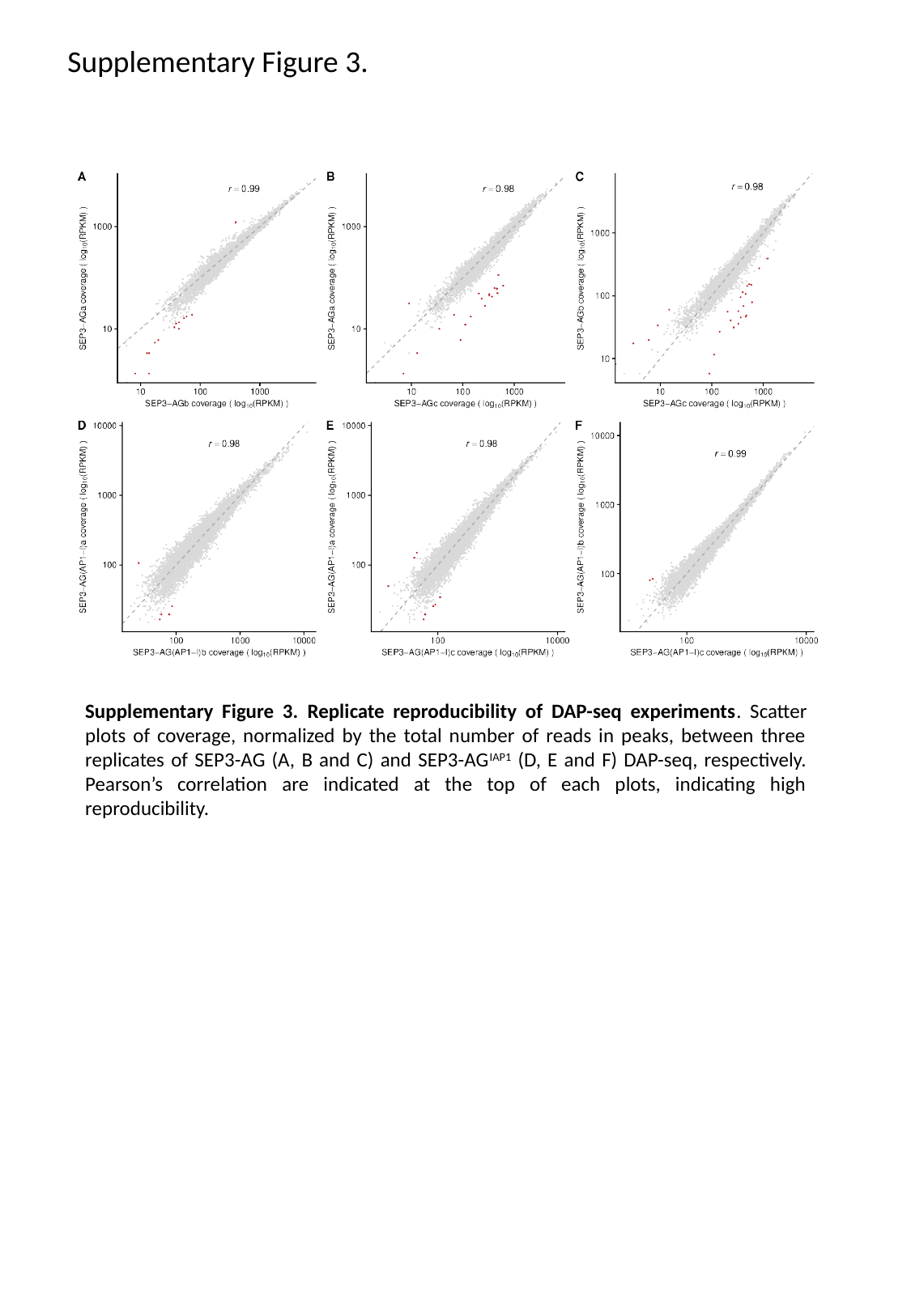

Supplementary Figure 3.
Supplementary Figure 3. Replicate reproducibility of DAP-seq experiments. Scatter plots of coverage, normalized by the total number of reads in peaks, between three replicates of SEP3-AG (A, B and C) and SEP3-AGIAP1 (D, E and F) DAP-seq, respectively. Pearson’s correlation are indicated at the top of each plots, indicating high reproducibility.

## Slide 6
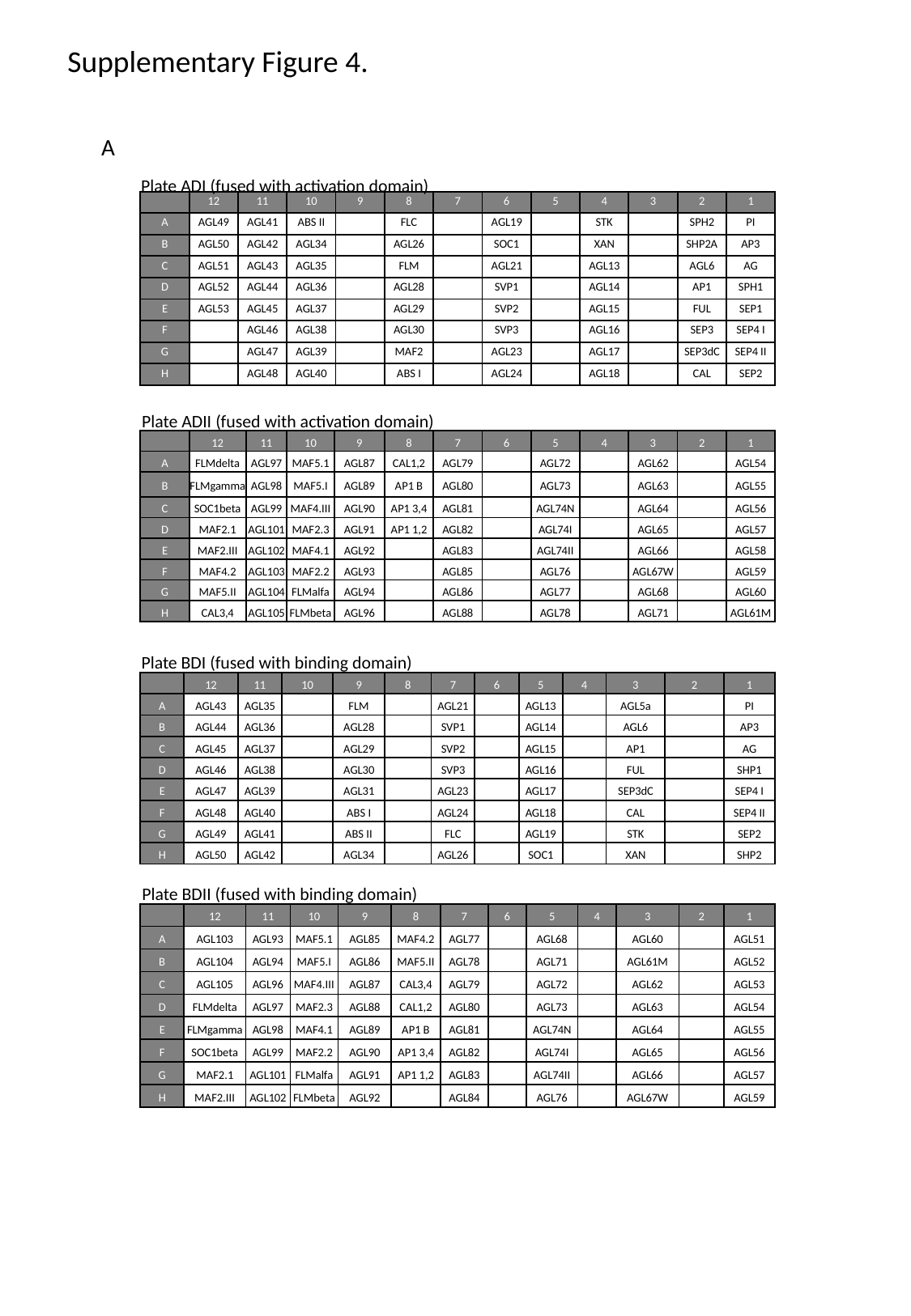

Supplementary Figure 4.
A
Plate ADI (fused with activation domain)
| | 12 | 11 | 10 | 9 | 8 | 7 | 6 | 5 | 4 | 3 | 2 | 1 |
| --- | --- | --- | --- | --- | --- | --- | --- | --- | --- | --- | --- | --- |
| A | AGL49 | AGL41 | ABS II | | FLC | | AGL19 | | STK | | SPH2 | PI |
| B | AGL50 | AGL42 | AGL34 | | AGL26 | | SOC1 | | XAN | | SHP2A | AP3 |
| C | AGL51 | AGL43 | AGL35 | | FLM | | AGL21 | | AGL13 | | AGL6 | AG |
| D | AGL52 | AGL44 | AGL36 | | AGL28 | | SVP1 | | AGL14 | | AP1 | SPH1 |
| E | AGL53 | AGL45 | AGL37 | | AGL29 | | SVP2 | | AGL15 | | FUL | SEP1 |
| F | | AGL46 | AGL38 | | AGL30 | | SVP3 | | AGL16 | | SEP3 | SEP4 I |
| G | | AGL47 | AGL39 | | MAF2 | | AGL23 | | AGL17 | | SEP3dC | SEP4 II |
| H | | AGL48 | AGL40 | | ABS I | | AGL24 | | AGL18 | | CAL | SEP2 |
Plate ADII (fused with activation domain)
| | 12 | 11 | 10 | 9 | 8 | 7 | 6 | 5 | 4 | 3 | 2 | 1 |
| --- | --- | --- | --- | --- | --- | --- | --- | --- | --- | --- | --- | --- |
| A | FLMdelta | AGL97 | MAF5.1 | AGL87 | CAL1,2 | AGL79 | | AGL72 | | AGL62 | | AGL54 |
| B | FLMgamma | AGL98 | MAF5.I | AGL89 | AP1 B | AGL80 | | AGL73 | | AGL63 | | AGL55 |
| C | SOC1beta | AGL99 | MAF4.III | AGL90 | AP1 3,4 | AGL81 | | AGL74N | | AGL64 | | AGL56 |
| D | MAF2.1 | AGL101 | MAF2.3 | AGL91 | AP1 1,2 | AGL82 | | AGL74I | | AGL65 | | AGL57 |
| E | MAF2.III | AGL102 | MAF4.1 | AGL92 | | AGL83 | | AGL74II | | AGL66 | | AGL58 |
| F | MAF4.2 | AGL103 | MAF2.2 | AGL93 | | AGL85 | | AGL76 | | AGL67W | | AGL59 |
| G | MAF5.II | AGL104 | FLMalfa | AGL94 | | AGL86 | | AGL77 | | AGL68 | | AGL60 |
| H | CAL3,4 | AGL105 | FLMbeta | AGL96 | | AGL88 | | AGL78 | | AGL71 | | AGL61M |
Plate BDI (fused with binding domain)
| | 12 | 11 | 10 | 9 | 8 | 7 | 6 | 5 | 4 | 3 | 2 | 1 |
| --- | --- | --- | --- | --- | --- | --- | --- | --- | --- | --- | --- | --- |
| A | AGL43 | AGL35 | | FLM | | AGL21 | | AGL13 | | AGL5a | | PI |
| B | AGL44 | AGL36 | | AGL28 | | SVP1 | | AGL14 | | AGL6 | | AP3 |
| C | AGL45 | AGL37 | | AGL29 | | SVP2 | | AGL15 | | AP1 | | AG |
| D | AGL46 | AGL38 | | AGL30 | | SVP3 | | AGL16 | | FUL | | SHP1 |
| E | AGL47 | AGL39 | | AGL31 | | AGL23 | | AGL17 | | SEP3dC | | SEP4 I |
| F | AGL48 | AGL40 | | ABS I | | AGL24 | | AGL18 | | CAL | | SEP4 II |
| G | AGL49 | AGL41 | | ABS II | | FLC | | AGL19 | | STK | | SEP2 |
| H | AGL50 | AGL42 | | AGL34 | | AGL26 | | SOC1 | | XAN | | SHP2 |
Plate BDII (fused with binding domain)
| | 12 | 11 | 10 | 9 | 8 | 7 | 6 | 5 | 4 | 3 | 2 | 1 |
| --- | --- | --- | --- | --- | --- | --- | --- | --- | --- | --- | --- | --- |
| A | AGL103 | AGL93 | MAF5.1 | AGL85 | MAF4.2 | AGL77 | | AGL68 | | AGL60 | | AGL51 |
| B | AGL104 | AGL94 | MAF5.I | AGL86 | MAF5.II | AGL78 | | AGL71 | | AGL61M | | AGL52 |
| C | AGL105 | AGL96 | MAF4.III | AGL87 | CAL3,4 | AGL79 | | AGL72 | | AGL62 | | AGL53 |
| D | FLMdelta | AGL97 | MAF2.3 | AGL88 | CAL1,2 | AGL80 | | AGL73 | | AGL63 | | AGL54 |
| E | FLMgamma | AGL98 | MAF4.1 | AGL89 | AP1 B | AGL81 | | AGL74N | | AGL64 | | AGL55 |
| F | SOC1beta | AGL99 | MAF2.2 | AGL90 | AP1 3,4 | AGL82 | | AGL74I | | AGL65 | | AGL56 |
| G | MAF2.1 | AGL101 | FLMalfa | AGL91 | AP1 1,2 | AGL83 | | AGL74II | | AGL66 | | AGL57 |
| H | MAF2.III | AGL102 | FLMbeta | AGL92 | | AGL84 | | AGL76 | | AGL67W | | AGL59 |

## Slide 7
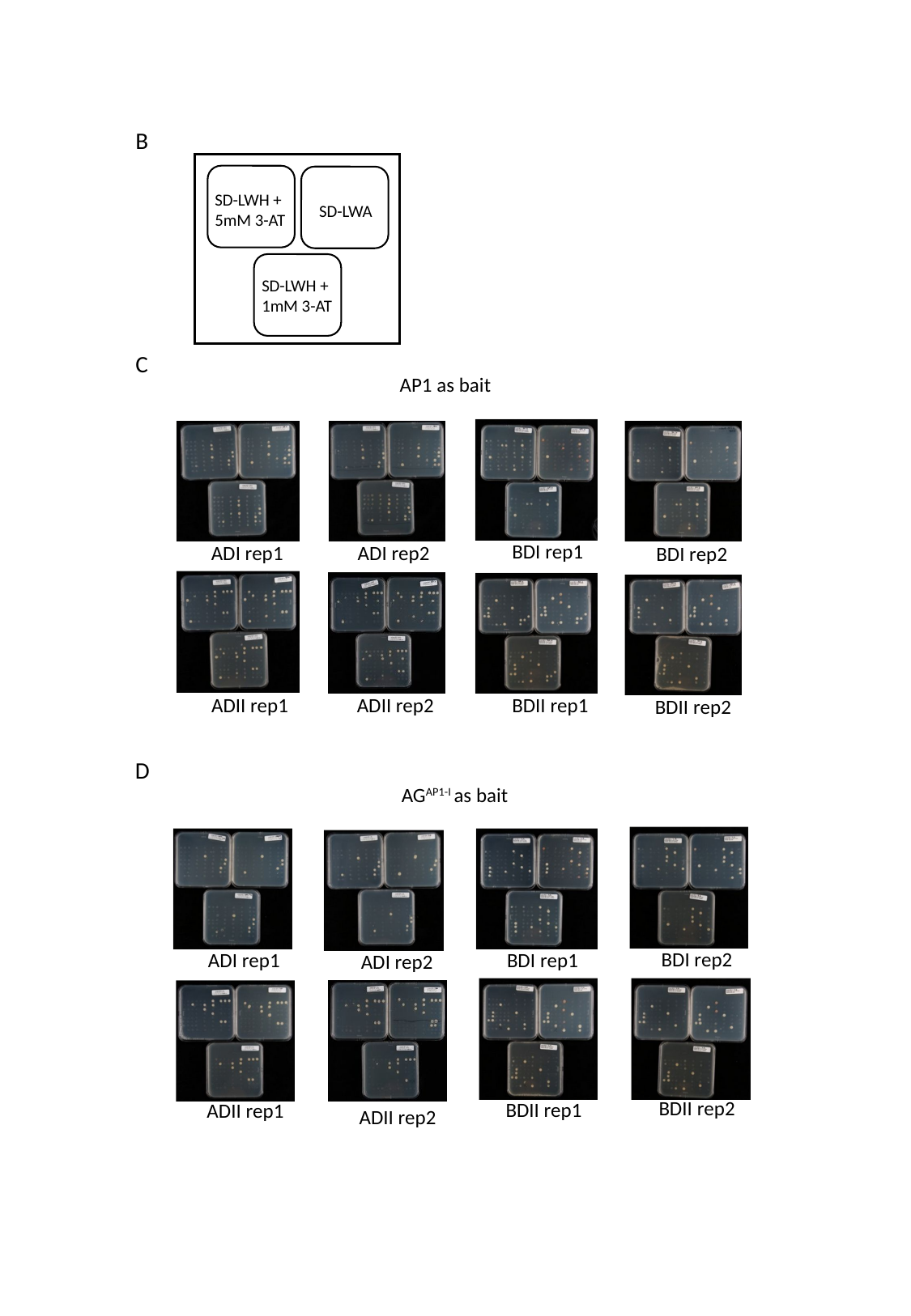

B
SD-LWH + 5mM 3-AT
SD-LWA
SD-LWH + 1mM 3-AT
C
AP1 as bait
BDI rep1
ADI rep2
ADI rep1
BDI rep2
ADII rep1
BDII rep1
ADII rep2
BDII rep2
D
AGAP1-I as bait
BDI rep2
BDI rep1
ADI rep1
ADI rep2
BDII rep2
BDII rep1
ADII rep1
ADII rep2

## Slide 8
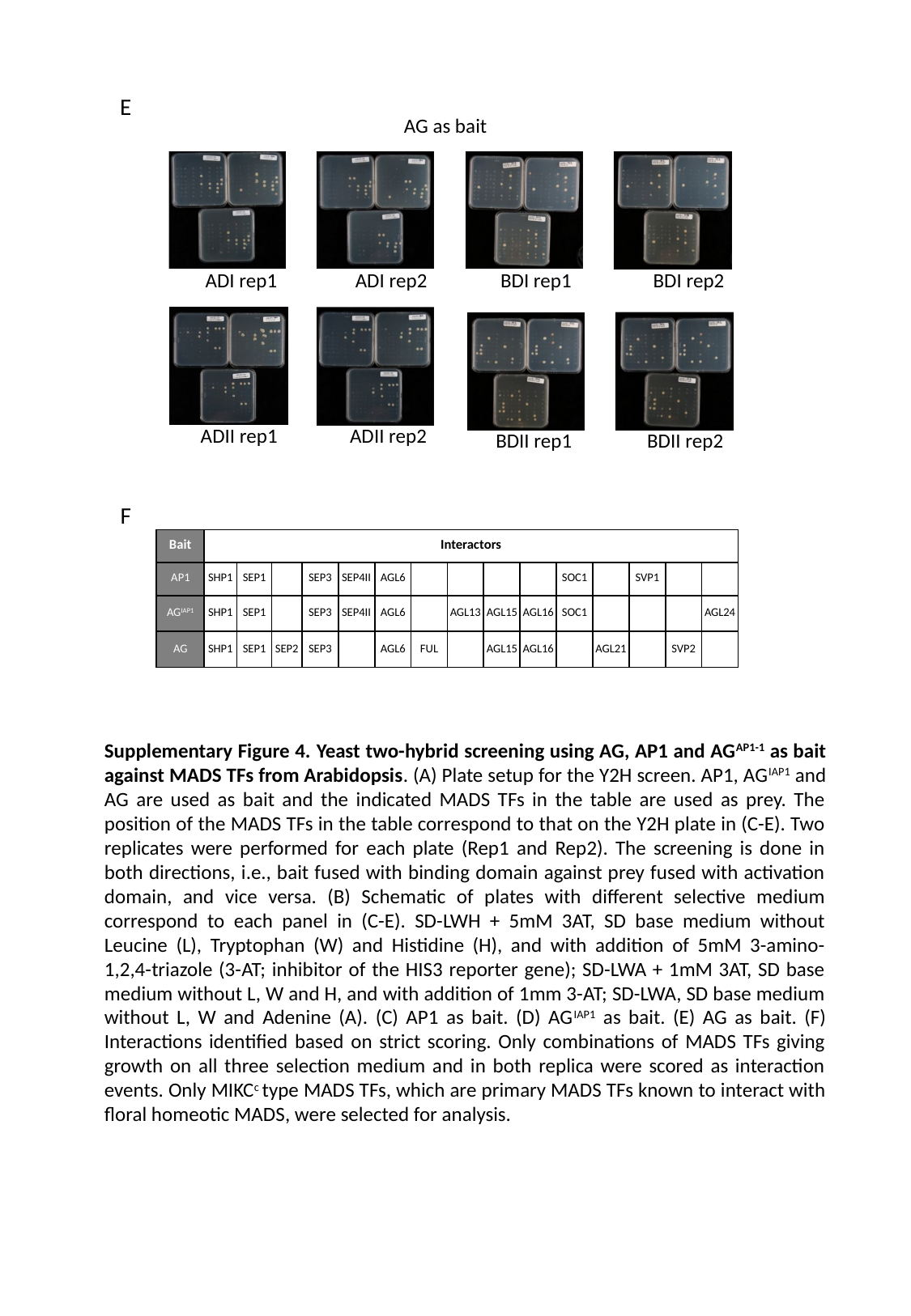

E
AG as bait
ADI rep2
BDI rep1
BDI rep2
ADI rep1
ADII rep1
ADII rep2
BDII rep1
BDII rep2
F
| Bait | Interactors | | | | | | | | | | | | | | |
| --- | --- | --- | --- | --- | --- | --- | --- | --- | --- | --- | --- | --- | --- | --- | --- |
| AP1 | SHP1 | SEP1 | | SEP3 | SEP4II | AGL6 | | | | | SOC1 | | SVP1 | | |
| AGIAP1 | SHP1 | SEP1 | | SEP3 | SEP4II | AGL6 | | AGL13 | AGL15 | AGL16 | SOC1 | | | | AGL24 |
| AG | SHP1 | SEP1 | SEP2 | SEP3 | | AGL6 | FUL | | AGL15 | AGL16 | | AGL21 | | SVP2 | |
Supplementary Figure 4. Yeast two-hybrid screening using AG, AP1 and AGAP1-1 as bait against MADS TFs from Arabidopsis. (A) Plate setup for the Y2H screen. AP1, AGIAP1 and AG are used as bait and the indicated MADS TFs in the table are used as prey. The position of the MADS TFs in the table correspond to that on the Y2H plate in (C-E). Two replicates were performed for each plate (Rep1 and Rep2). The screening is done in both directions, i.e., bait fused with binding domain against prey fused with activation domain, and vice versa. (B) Schematic of plates with different selective medium correspond to each panel in (C-E). SD-LWH + 5mM 3AT, SD base medium without Leucine (L), Tryptophan (W) and Histidine (H), and with addition of 5mM 3-amino-1,2,4-triazole (3-AT; inhibitor of the HIS3 reporter gene); SD-LWA + 1mM 3AT, SD base medium without L, W and H, and with addition of 1mm 3-AT; SD-LWA, SD base medium without L, W and Adenine (A). (C) AP1 as bait. (D) AGIAP1 as bait. (E) AG as bait. (F) Interactions identified based on strict scoring. Only combinations of MADS TFs giving growth on all three selection medium and in both replica were scored as interaction events. Only MIKCc type MADS TFs, which are primary MADS TFs known to interact with floral homeotic MADS, were selected for analysis.

## Slide 9
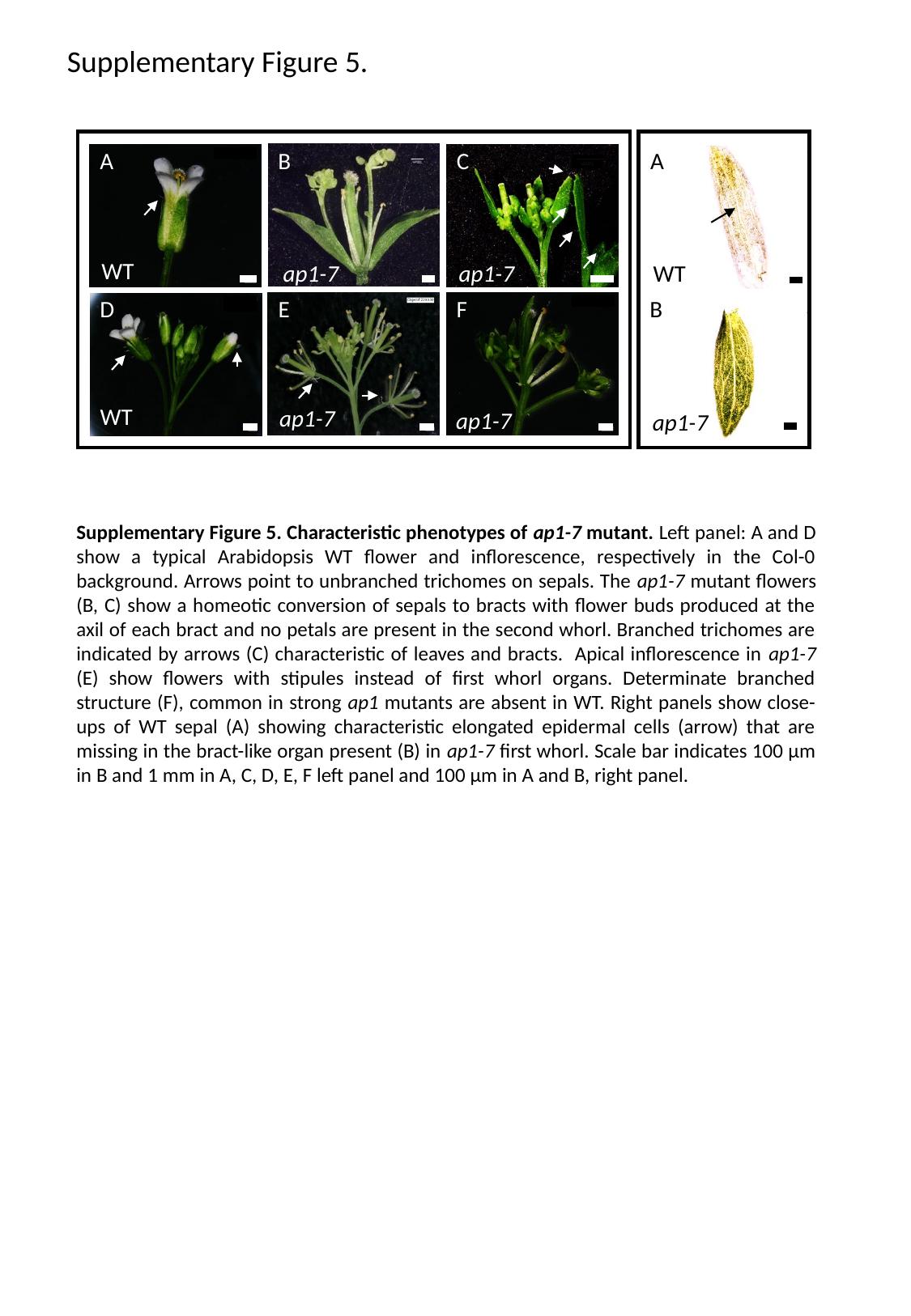

Supplementary Figure 5.
A
B
C
A
ap1-
WT
ap1-7
ap1-7
WT
D
E
F
B
WT
ap1-7
ap1-7
ap1-7
Supplementary Figure 5. Characteristic phenotypes of ap1-7 mutant. Left panel: A and D show a typical Arabidopsis WT flower and inflorescence, respectively in the Col-0 background. Arrows point to unbranched trichomes on sepals. The ap1-7 mutant flowers (B, C) show a homeotic conversion of sepals to bracts with flower buds produced at the axil of each bract and no petals are present in the second whorl. Branched trichomes are indicated by arrows (C) characteristic of leaves and bracts. Apical inflorescence in ap1-7 (E) show flowers with stipules instead of first whorl organs. Determinate branched structure (F), common in strong ap1 mutants are absent in WT. Right panels show close-ups of WT sepal (A) showing characteristic elongated epidermal cells (arrow) that are missing in the bract-like organ present (B) in ap1-7 first whorl. Scale bar indicates 100 µm in B and 1 mm in A, C, D, E, F left panel and 100 µm in A and B, right panel.

## Slide 10
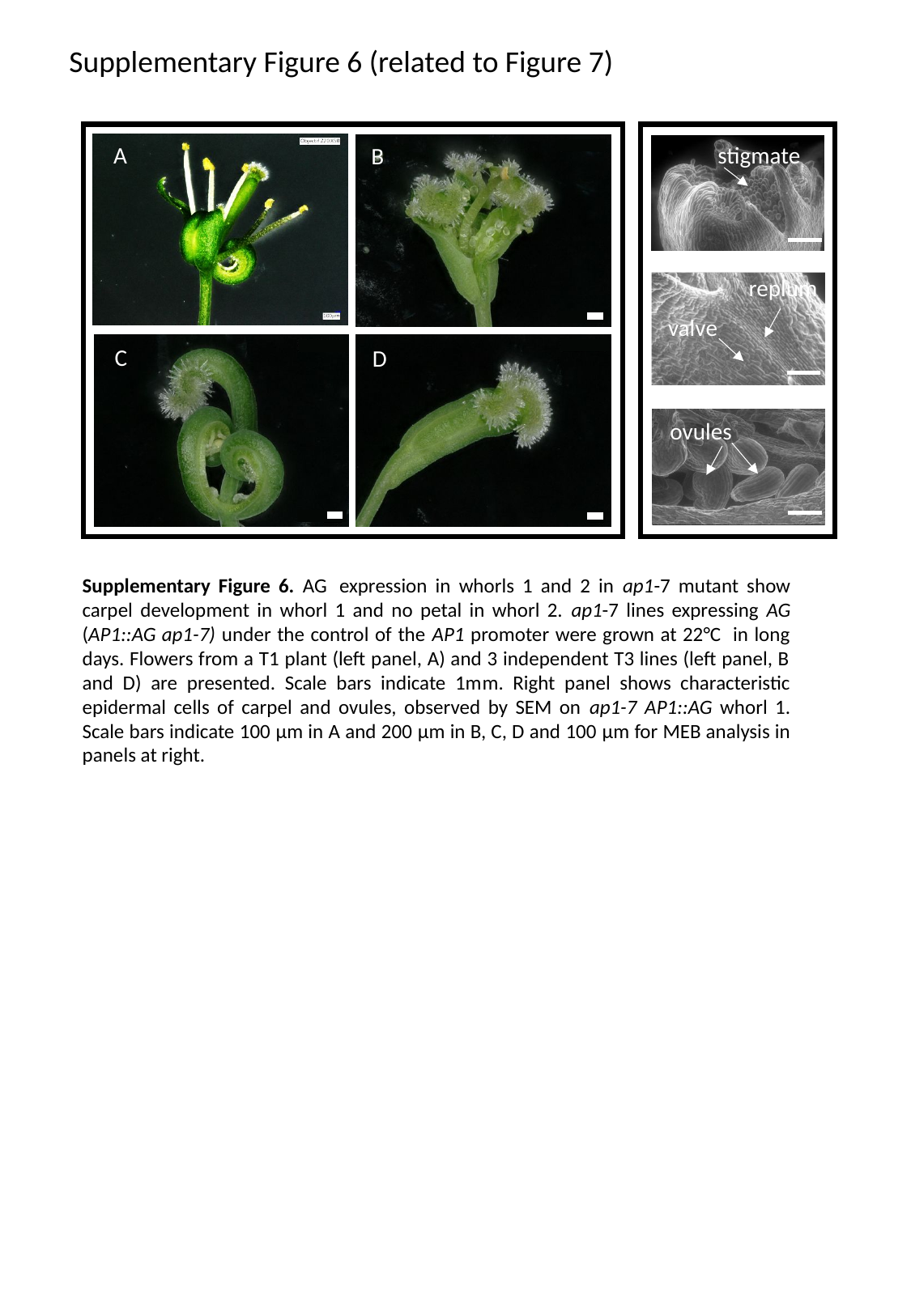

Supplementary Figure 6 (related to Figure 7)
A
stigmate
replum
valve
ovules
B
WT
C
D
# 7.4
Supplementary Figure 6. AG expression in whorls 1 and 2 in ap1-7 mutant show carpel development in whorl 1 and no petal in whorl 2. ap1-7 lines expressing AG (AP1::AG ap1-7) under the control of the AP1 promoter were grown at 22°C in long days. Flowers from a T1 plant (left panel, A) and 3 independent T3 lines (left panel, B and D) are presented. Scale bars indicate 1mm. Right panel shows characteristic epidermal cells of carpel and ovules, observed by SEM on ap1-7 AP1::AG whorl 1. Scale bars indicate 100 µm in A and 200 µm in B, C, D and 100 µm for MEB analysis in panels at right.
ap1-7
AP1::AG

## Slide 11
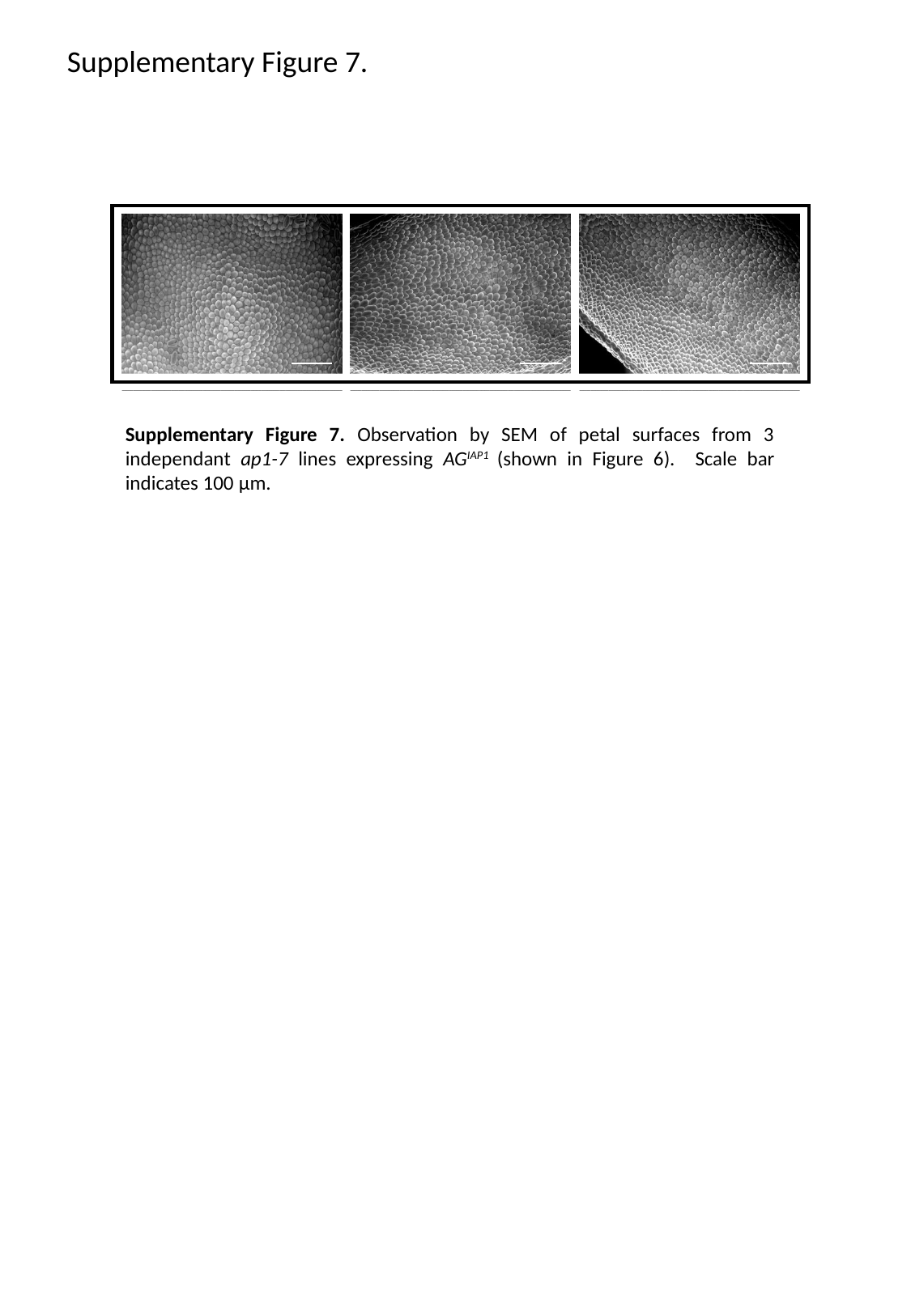

Supplementary Figure 7.
Supplementary Figure 7. Observation by SEM of petal surfaces from 3 independant ap1-7 lines expressing AGIAP1 (shown in Figure 6). Scale bar indicates 100 µm.

## Slide 12
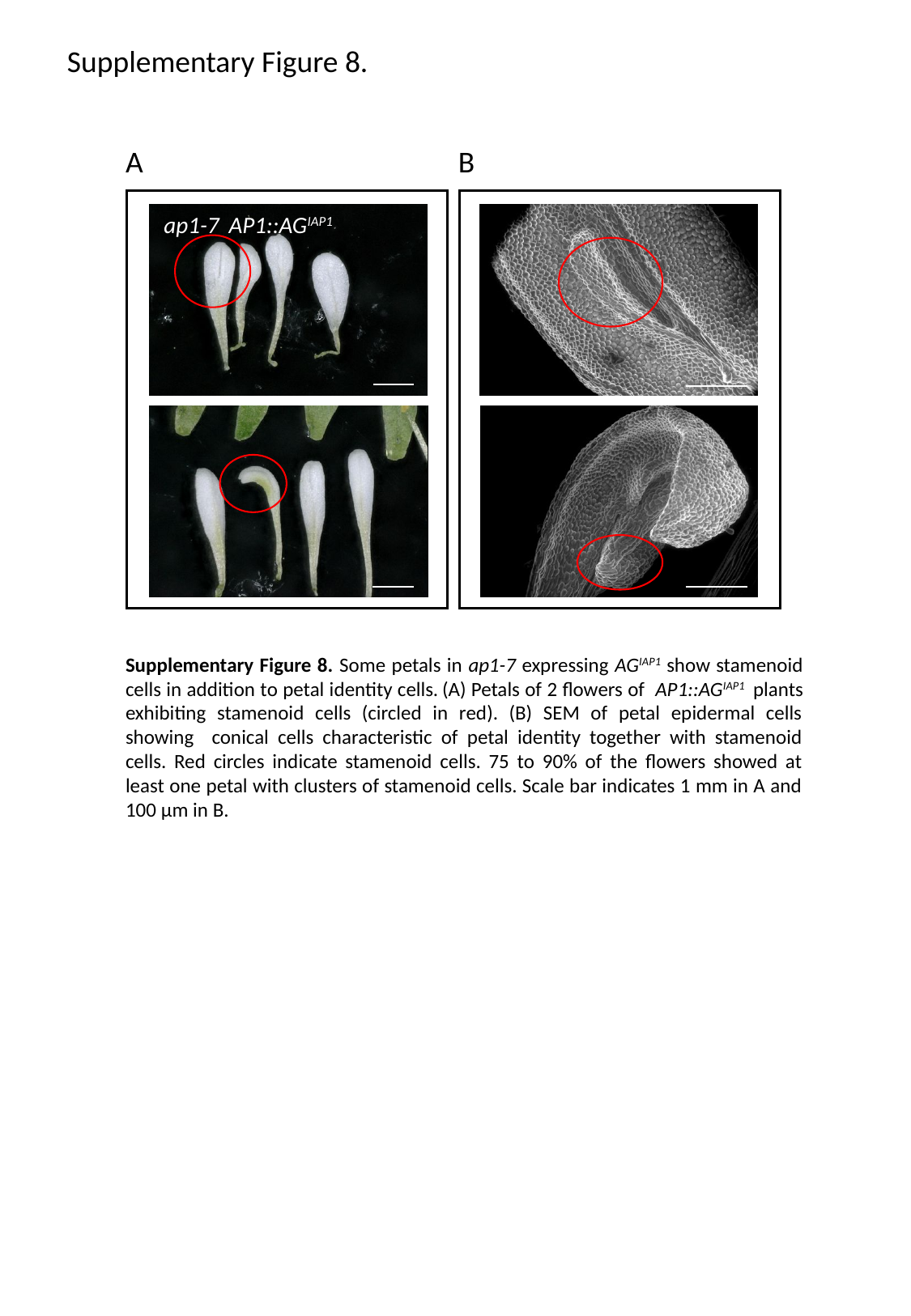

Supplementary Figure 8.
A
B
ap1-7
AP1::AGIAP1
Supplementary Figure 8. Some petals in ap1-7 expressing AGIAP1 show stamenoid cells in addition to petal identity cells. (A) Petals of 2 flowers of AP1::AGIAP1 plants exhibiting stamenoid cells (circled in red). (B) SEM of petal epidermal cells showing conical cells characteristic of petal identity together with stamenoid cells. Red circles indicate stamenoid cells. 75 to 90% of the flowers showed at least one petal with clusters of stamenoid cells. Scale bar indicates 1 mm in A and 100 µm in B.

## Slide 13
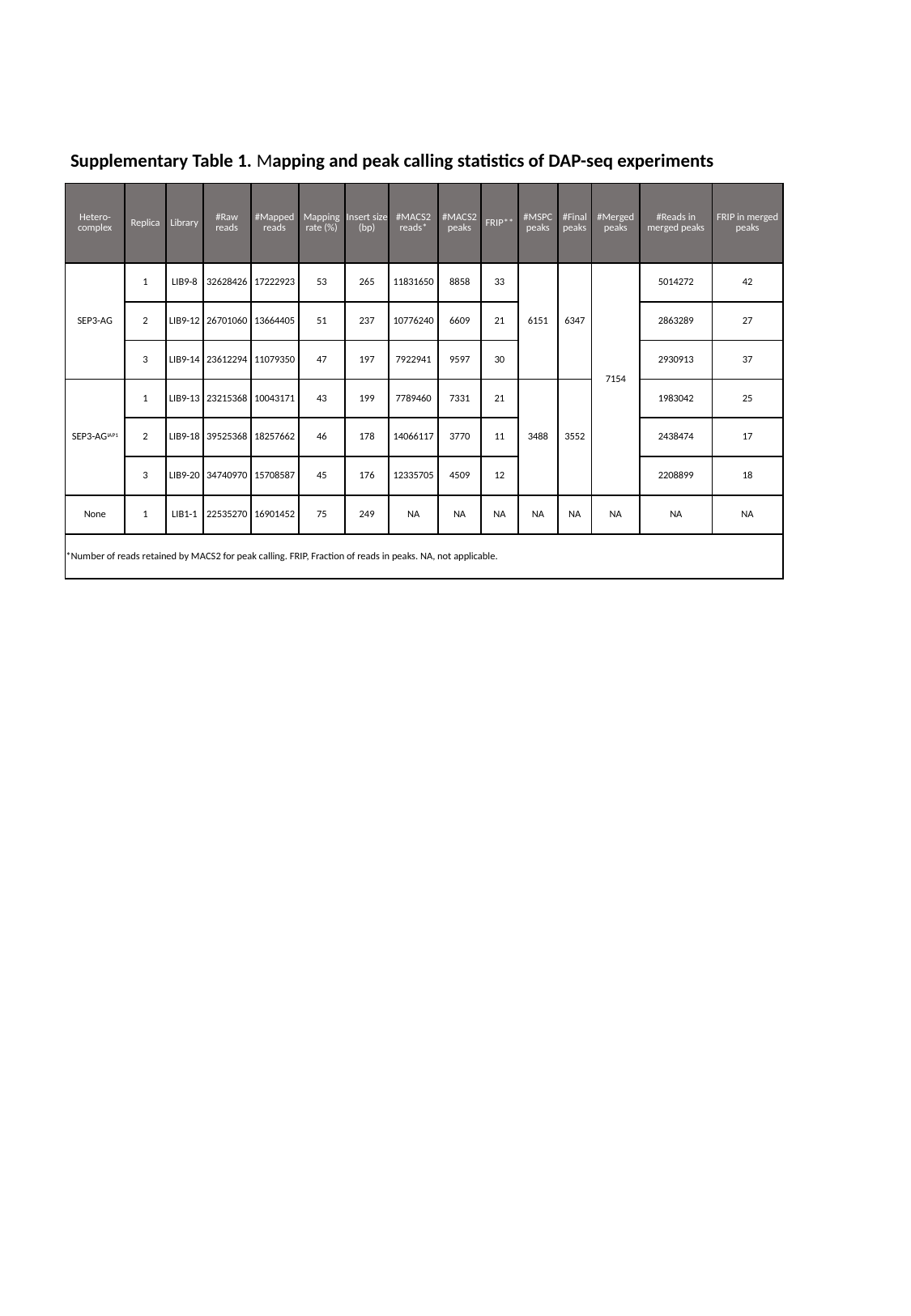

Supplementary Table 1. Mapping and peak calling statistics of DAP-seq experiments
| Hetero-complex | Replica | Library | #Raw reads | #Mapped reads | Mapping rate (%) | Insert size (bp) | #MACS2 reads\* | #MACS2 peaks | FRIP\*\* | #MSPC peaks | #Final peaks | #Merged peaks | #Reads in merged peaks | FRIP in merged peaks |
| --- | --- | --- | --- | --- | --- | --- | --- | --- | --- | --- | --- | --- | --- | --- |
| SEP3-AG | 1 | LIB9-8 | 32628426 | 17222923 | 53 | 265 | 11831650 | 8858 | 33 | 6151 | 6347 | 7154 | 5014272 | 42 |
| | 2 | LIB9-12 | 26701060 | 13664405 | 51 | 237 | 10776240 | 6609 | 21 | | | | 2863289 | 27 |
| | 3 | LIB9-14 | 23612294 | 11079350 | 47 | 197 | 7922941 | 9597 | 30 | | | | 2930913 | 37 |
| SEP3-AGIAP1 | 1 | LIB9-13 | 23215368 | 10043171 | 43 | 199 | 7789460 | 7331 | 21 | 3488 | 3552 | | 1983042 | 25 |
| | 2 | LIB9-18 | 39525368 | 18257662 | 46 | 178 | 14066117 | 3770 | 11 | | | | 2438474 | 17 |
| | 3 | LIB9-20 | 34740970 | 15708587 | 45 | 176 | 12335705 | 4509 | 12 | | | | 2208899 | 18 |
| None | 1 | LIB1-1 | 22535270 | 16901452 | 75 | 249 | NA | NA | NA | NA | NA | NA | NA | NA |
| \*Number of reads retained by MACS2 for peak calling. FRIP, Fraction of reads in peaks. NA, not applicable. | | | | | | | | | | | | | | |

## Slide 14
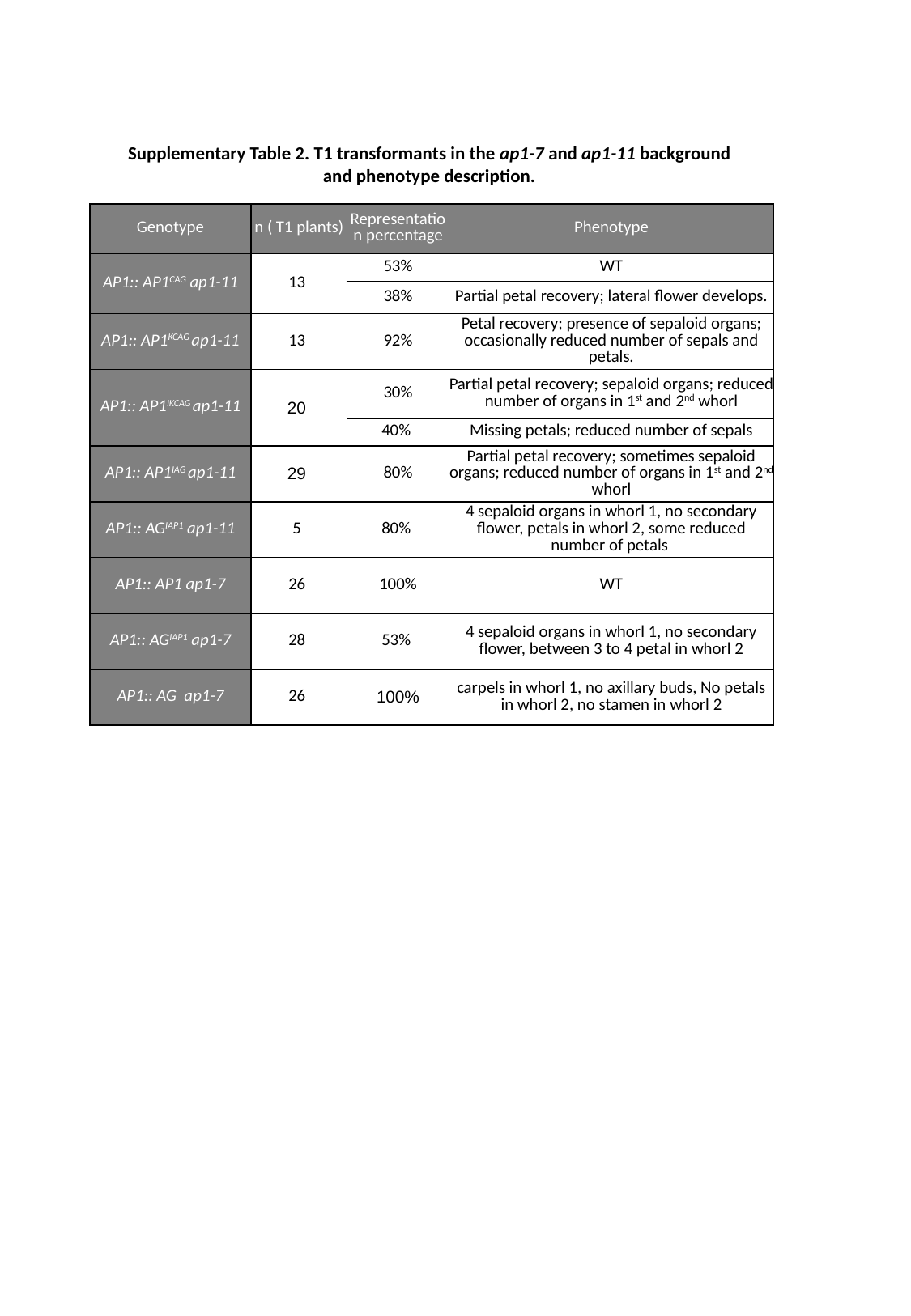

Supplementary Table 2. T1 transformants in the ap1-7 and ap1-11 background
and phenotype description.
| Genotype | n ( T1 plants) | Representation percentage | Phenotype |
| --- | --- | --- | --- |
| AP1:: AP1CAG ap1-11 | 13 | 53% | WT |
| | | 38% | Partial petal recovery; lateral flower develops. |
| AP1:: AP1KCAG ap1-11 | 13 | 92% | Petal recovery; presence of sepaloid organs; occasionally reduced number of sepals and petals. |
| AP1:: AP1IKCAG ap1-11 | 20 | 30% | Partial petal recovery; sepaloid organs; reduced number of organs in 1st and 2nd whorl |
| | | 40% | Missing petals; reduced number of sepals |
| AP1:: AP1IAG ap1-11 | 29 | 80% | Partial petal recovery; sometimes sepaloid organs; reduced number of organs in 1st and 2nd whorl |
| AP1:: AGIAP1 ap1-11 | 5 | 80% | 4 sepaloid organs in whorl 1, no secondary flower, petals in whorl 2, some reduced number of petals |
| AP1:: AP1 ap1-7 | 26 | 100% | WT |
| AP1:: AGIAP1 ap1-7 | 28 | 53% | 4 sepaloid organs in whorl 1, no secondary flower, between 3 to 4 petal in whorl 2 |
| AP1:: AG ap1-7 | 26 | 100% | carpels in whorl 1, no axillary buds, No petals in whorl 2, no stamen in whorl 2 |

## Slide 15
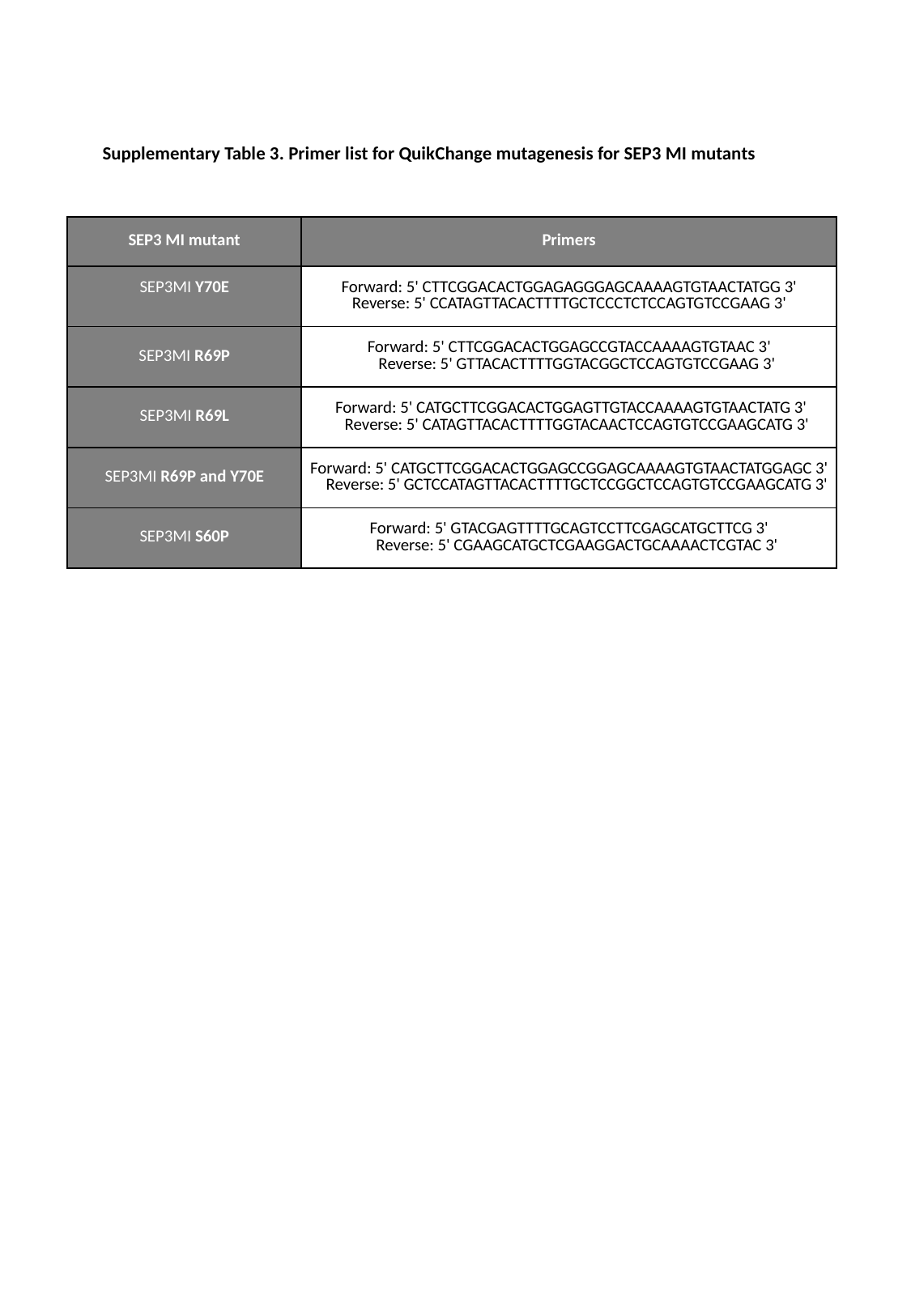

Supplementary Table 3. Primer list for QuikChange mutagenesis for SEP3 MI mutants
| SEP3 MI mutant | Primers |
| --- | --- |
| SEP3MI Y70E | Forward: 5' CTTCGGACACTGGAGAGGGAGCAAAAGTGTAACTATGG 3' Reverse: 5' CCATAGTTACACTTTTGCTCCCTCTCCAGTGTCCGAAG 3' |
| SEP3MI R69P | Forward: 5' CTTCGGACACTGGAGCCGTACCAAAAGTGTAAC 3'     Reverse: 5' GTTACACTTTTGGTACGGCTCCAGTGTCCGAAG 3' |
| SEP3MI R69L | Forward: 5' CATGCTTCGGACACTGGAGTTGTACCAAAAGTGTAACTATG 3'     Reverse: 5' CATAGTTACACTTTTGGTACAACTCCAGTGTCCGAAGCATG 3' |
| SEP3MI R69P and Y70E | Forward: 5' CATGCTTCGGACACTGGAGCCGGAGCAAAAGTGTAACTATGGAGC 3'     Reverse: 5' GCTCCATAGTTACACTTTTGCTCCGGCTCCAGTGTCCGAAGCATG 3' |
| SEP3MI S60P | Forward: 5' GTACGAGTTTTGCAGTCCTTCGAGCATGCTTCG 3'     Reverse: 5' CGAAGCATGCTCGAAGGACTGCAAAACTCGTAC 3' |

## Slide 16
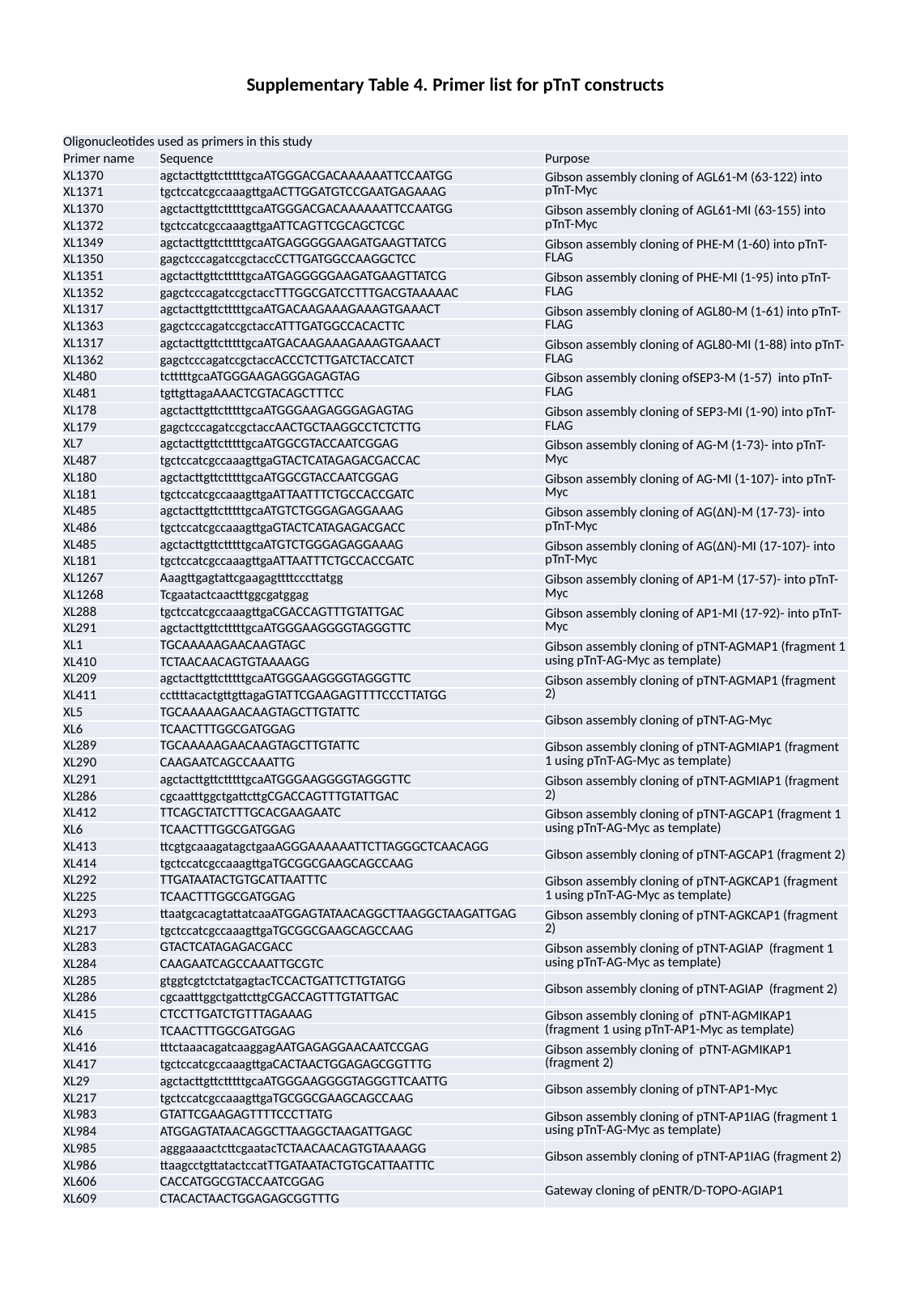

Supplementary Table 4. Primer list for pTnT constructs
| Oligonucleotides used as primers in this study | | |
| --- | --- | --- |
| Primer name | Sequence | Purpose |
| XL1370 | agctacttgttctttttgcaATGGGACGACAAAAAATTCCAATGG | Gibson assembly cloning of AGL61-M (63-122) into pTnT-Myc |
| XL1371 | tgctccatcgccaaagttgaACTTGGATGTCCGAATGAGAAAG | |
| XL1370 | agctacttgttctttttgcaATGGGACGACAAAAAATTCCAATGG | Gibson assembly cloning of AGL61-MI (63-155) into pTnT-Myc |
| XL1372 | tgctccatcgccaaagttgaATTCAGTTCGCAGCTCGC | |
| XL1349 | agctacttgttctttttgcaATGAGGGGGAAGATGAAGTTATCG | Gibson assembly cloning of PHE-M (1-60) into pTnT-FLAG |
| XL1350 | gagctcccagatccgctaccCCTTGATGGCCAAGGCTCC | |
| XL1351 | agctacttgttctttttgcaATGAGGGGGAAGATGAAGTTATCG | Gibson assembly cloning of PHE-MI (1-95) into pTnT-FLAG |
| XL1352 | gagctcccagatccgctaccTTTGGCGATCCTTTGACGTAAAAAC | |
| XL1317 | agctacttgttctttttgcaATGACAAGAAAGAAAGTGAAACT | Gibson assembly cloning of AGL80-M (1-61) into pTnT-FLAG |
| XL1363 | gagctcccagatccgctaccATTTGATGGCCACACTTC | |
| XL1317 | agctacttgttctttttgcaATGACAAGAAAGAAAGTGAAACT | Gibson assembly cloning of AGL80-MI (1-88) into pTnT-FLAG |
| XL1362 | gagctcccagatccgctaccACCCTCTTGATCTACCATCT | |
| XL480 | tctttttgcaATGGGAAGAGGGAGAGTAG | Gibson assembly cloning ofSEP3-M (1-57) into pTnT-FLAG |
| XL481 | tgttgttagaAAACTCGTACAGCTTTCC | |
| XL178 | agctacttgttctttttgcaATGGGAAGAGGGAGAGTAG | Gibson assembly cloning of SEP3-MI (1-90) into pTnT-FLAG |
| XL179 | gagctcccagatccgctaccAACTGCTAAGGCCTCTCTTG | |
| XL7 | agctacttgttctttttgcaATGGCGTACCAATCGGAG | Gibson assembly cloning of AG-M (1-73)- into pTnT-Myc |
| XL487 | tgctccatcgccaaagttgaGTACTCATAGAGACGACCAC | |
| XL180 | agctacttgttctttttgcaATGGCGTACCAATCGGAG | Gibson assembly cloning of AG-MI (1-107)- into pTnT-Myc |
| XL181 | tgctccatcgccaaagttgaATTAATTTCTGCCACCGATC | |
| XL485 | agctacttgttctttttgcaATGTCTGGGAGAGGAAAG | Gibson assembly cloning of AG(ΔN)-M (17-73)- into pTnT-Myc |
| XL486 | tgctccatcgccaaagttgaGTACTCATAGAGACGACC | |
| XL485 | agctacttgttctttttgcaATGTCTGGGAGAGGAAAG | Gibson assembly cloning of AG(ΔN)-MI (17-107)- into pTnT-Myc |
| XL181 | tgctccatcgccaaagttgaATTAATTTCTGCCACCGATC | |
| XL1267 | Aaagttgagtattcgaagagttttcccttatgg | Gibson assembly cloning of AP1-M (17-57)- into pTnT-Myc |
| XL1268 | Tcgaatactcaactttggcgatggag | |
| XL288 | tgctccatcgccaaagttgaCGACCAGTTTGTATTGAC | Gibson assembly cloning of AP1-MI (17-92)- into pTnT-Myc |
| XL291 | agctacttgttctttttgcaATGGGAAGGGGTAGGGTTC | |
| XL1 | TGCAAAAAGAACAAGTAGC | Gibson assembly cloning of pTNT-AGMAP1 (fragment 1 using pTnT-AG-Myc as template) |
| XL410 | TCTAACAACAGTGTAAAAGG | |
| XL209 | agctacttgttctttttgcaATGGGAAGGGGTAGGGTTC | Gibson assembly cloning of pTNT-AGMAP1 (fragment 2) |
| XL411 | ccttttacactgttgttagaGTATTCGAAGAGTTTTCCCTTATGG | |
| XL5 | TGCAAAAAGAACAAGTAGCTTGTATTC | Gibson assembly cloning of pTNT-AG-Myc |
| XL6 | TCAACTTTGGCGATGGAG | |
| XL289 | TGCAAAAAGAACAAGTAGCTTGTATTC | Gibson assembly cloning of pTNT-AGMIAP1 (fragment 1 using pTnT-AG-Myc as template) |
| XL290 | CAAGAATCAGCCAAATTG | |
| XL291 | agctacttgttctttttgcaATGGGAAGGGGTAGGGTTC | Gibson assembly cloning of pTNT-AGMIAP1 (fragment 2) |
| XL286 | cgcaatttggctgattcttgCGACCAGTTTGTATTGAC | |
| XL412 | TTCAGCTATCTTTGCACGAAGAATC | Gibson assembly cloning of pTNT-AGCAP1 (fragment 1 using pTnT-AG-Myc as template) |
| XL6 | TCAACTTTGGCGATGGAG | |
| XL413 | ttcgtgcaaagatagctgaaAGGGAAAAAATTCTTAGGGCTCAACAGG | Gibson assembly cloning of pTNT-AGCAP1 (fragment 2) |
| XL414 | tgctccatcgccaaagttgaTGCGGCGAAGCAGCCAAG | |
| XL292 | TTGATAATACTGTGCATTAATTTC | Gibson assembly cloning of pTNT-AGKCAP1 (fragment 1 using pTnT-AG-Myc as template) |
| XL225 | TCAACTTTGGCGATGGAG | |
| XL293 | ttaatgcacagtattatcaaATGGAGTATAACAGGCTTAAGGCTAAGATTGAG | Gibson assembly cloning of pTNT-AGKCAP1 (fragment 2) |
| XL217 | tgctccatcgccaaagttgaTGCGGCGAAGCAGCCAAG | |
| XL283 | GTACTCATAGAGACGACC | Gibson assembly cloning of pTNT-AGIAP (fragment 1 using pTnT-AG-Myc as template) |
| XL284 | CAAGAATCAGCCAAATTGCGTC | |
| XL285 | gtggtcgtctctatgagtacTCCACTGATTCTTGTATGG | Gibson assembly cloning of pTNT-AGIAP (fragment 2) |
| XL286 | cgcaatttggctgattcttgCGACCAGTTTGTATTGAC | |
| XL415 | CTCCTTGATCTGTTTAGAAAG | Gibson assembly cloning of pTNT-AGMIKAP1 (fragment 1 using pTnT-AP1-Myc as template) |
| XL6 | TCAACTTTGGCGATGGAG | |
| XL416 | tttctaaacagatcaaggagAATGAGAGGAACAATCCGAG | Gibson assembly cloning of pTNT-AGMIKAP1 (fragment 2) |
| XL417 | tgctccatcgccaaagttgaCACTAACTGGAGAGCGGTTTG | |
| XL29 | agctacttgttctttttgcaATGGGAAGGGGTAGGGTTCAATTG | Gibson assembly cloning of pTNT-AP1-Myc |
| XL217 | tgctccatcgccaaagttgaTGCGGCGAAGCAGCCAAG | |
| XL983 | GTATTCGAAGAGTTTTCCCTTATG | Gibson assembly cloning of pTNT-AP1IAG (fragment 1 using pTnT-AG-Myc as template) |
| XL984 | ATGGAGTATAACAGGCTTAAGGCTAAGATTGAGC | |
| XL985 | agggaaaactcttcgaatacTCTAACAACAGTGTAAAAGG | Gibson assembly cloning of pTNT-AP1IAG (fragment 2) |
| XL986 | ttaagcctgttatactccatTTGATAATACTGTGCATTAATTTC | |
| XL606 | CACCATGGCGTACCAATCGGAG | Gateway cloning of pENTR/D-TOPO-AGIAP1 |
| XL609 | CTACACTAACTGGAGAGCGGTTTG | |

## Slide 17
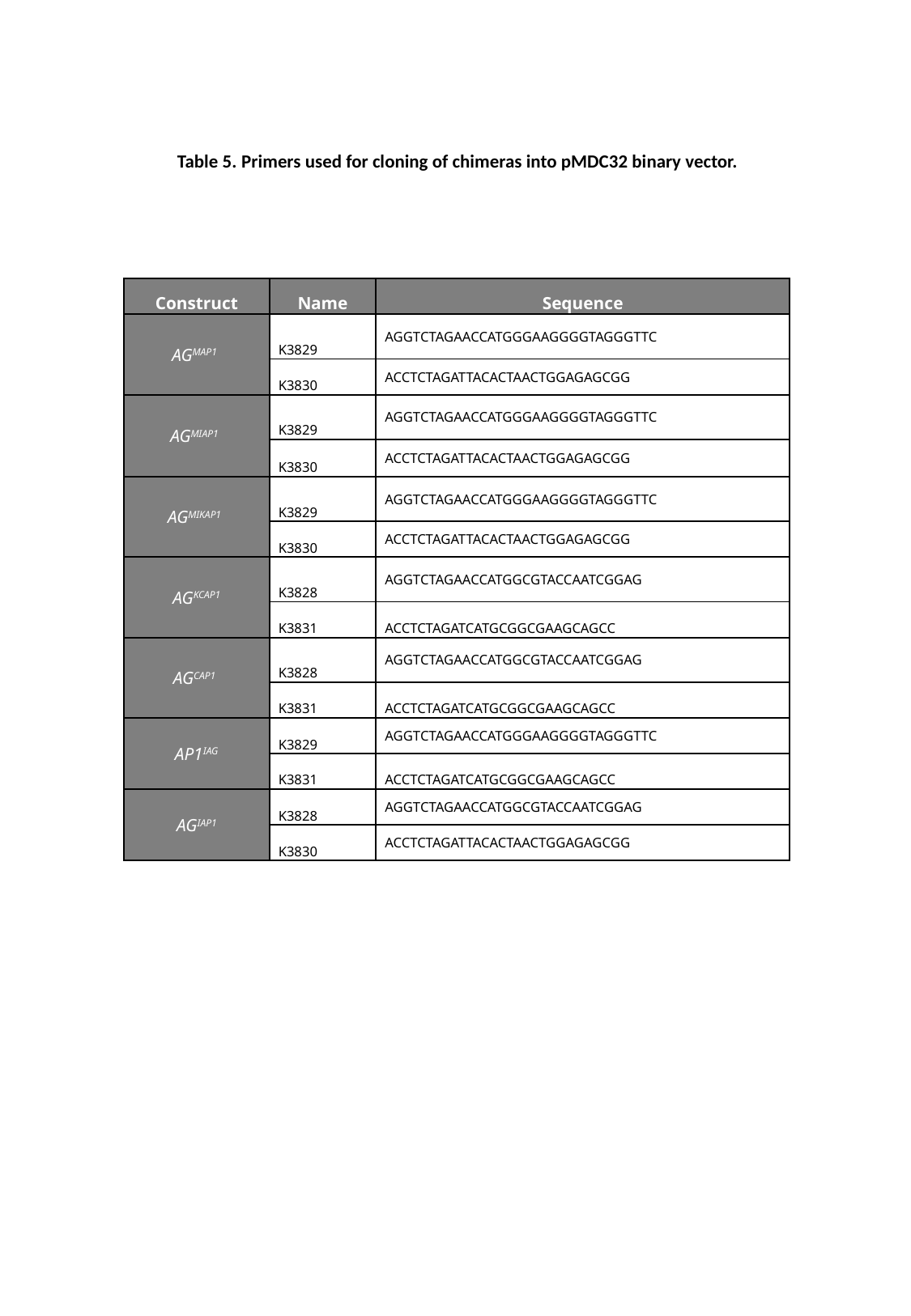

Table 5. Primers used for cloning of chimeras into pMDC32 binary vector.
| Construct | Name | Sequence |
| --- | --- | --- |
| AGMAP1 | K3829 | AGGTCTAGAACCATGGGAAGGGGTAGGGTTC |
| | K3830 | ACCTCTAGATTACACTAACTGGAGAGCGG |
| AGMIAP1 | K3829 | AGGTCTAGAACCATGGGAAGGGGTAGGGTTC |
| | K3830 | ACCTCTAGATTACACTAACTGGAGAGCGG |
| AGMIKAP1 | K3829 | AGGTCTAGAACCATGGGAAGGGGTAGGGTTC |
| | K3830 | ACCTCTAGATTACACTAACTGGAGAGCGG |
| AGKCAP1 | K3828 | AGGTCTAGAACCATGGCGTACCAATCGGAG |
| | K3831 | ACCTCTAGATCATGCGGCGAAGCAGCC |
| AGCAP1 | K3828 | AGGTCTAGAACCATGGCGTACCAATCGGAG |
| | K3831 | ACCTCTAGATCATGCGGCGAAGCAGCC |
| AP1IAG | K3829 | AGGTCTAGAACCATGGGAAGGGGTAGGGTTC |
| | K3831 | ACCTCTAGATCATGCGGCGAAGCAGCC |
| AGIAP1 | K3828 | AGGTCTAGAACCATGGCGTACCAATCGGAG |
| | K3830 | ACCTCTAGATTACACTAACTGGAGAGCGG |

## Slide 18
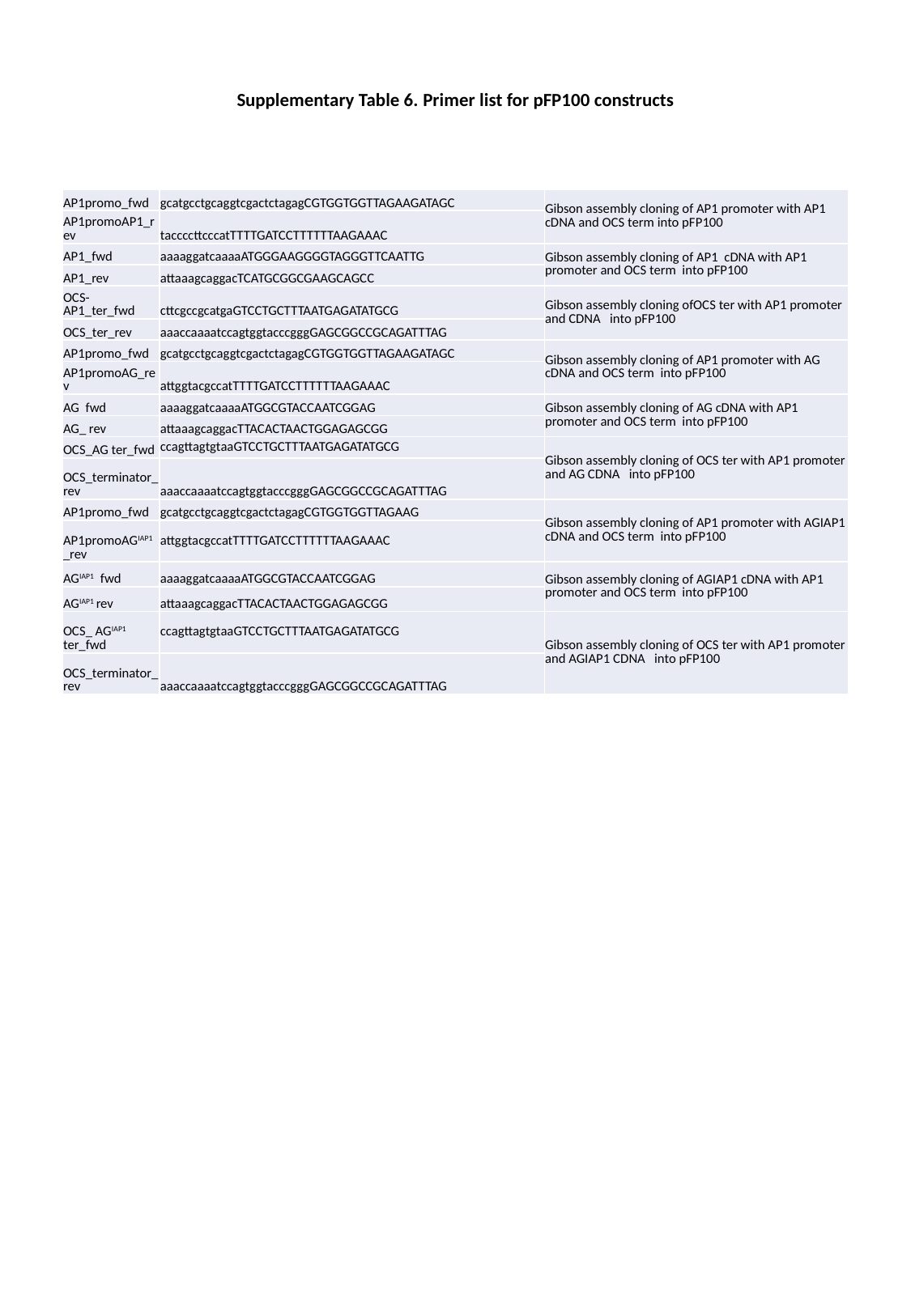

Supplementary Table 6. Primer list for pFP100 constructs
| AP1promo\_fwd | gcatgcctgcaggtcgactctagagCGTGGTGGTTAGAAGATAGC | Gibson assembly cloning of AP1 promoter with AP1 cDNA and OCS term into pFP100 |
| --- | --- | --- |
| AP1promoAP1\_rev | taccccttcccatTTTTGATCCTTTTTTAAGAAAC | |
| AP1\_fwd | aaaaggatcaaaaATGGGAAGGGGTAGGGTTCAATTG | Gibson assembly cloning of AP1 cDNA with AP1 promoter and OCS term into pFP100 |
| AP1\_rev | attaaagcaggacTCATGCGGCGAAGCAGCC | |
| OCS-AP1\_ter\_fwd | cttcgccgcatgaGTCCTGCTTTAATGAGATATGCG | Gibson assembly cloning ofOCS ter with AP1 promoter and CDNA into pFP100 |
| OCS\_ter\_rev | aaaccaaaatccagtggtacccgggGAGCGGCCGCAGATTTAG | |
| AP1promo\_fwd | gcatgcctgcaggtcgactctagagCGTGGTGGTTAGAAGATAGC | Gibson assembly cloning of AP1 promoter with AG cDNA and OCS term into pFP100 |
| AP1promoAG\_rev | attggtacgccatTTTTGATCCTTTTTTAAGAAAC | |
| AG fwd | aaaaggatcaaaaATGGCGTACCAATCGGAG | Gibson assembly cloning of AG cDNA with AP1 promoter and OCS term into pFP100 |
| AG\_ rev | attaaagcaggacTTACACTAACTGGAGAGCGG | |
| OCS\_AG ter\_fwd | ccagttagtgtaaGTCCTGCTTTAATGAGATATGCG | Gibson assembly cloning of OCS ter with AP1 promoter and AG CDNA into pFP100 |
| OCS\_terminator\_rev | aaaccaaaatccagtggtacccgggGAGCGGCCGCAGATTTAG | |
| AP1promo\_fwd | gcatgcctgcaggtcgactctagagCGTGGTGGTTAGAAG | Gibson assembly cloning of AP1 promoter with AGIAP1 cDNA and OCS term into pFP100 |
| AP1promoAGIAP1\_rev | attggtacgccatTTTTGATCCTTTTTTAAGAAAC | |
| AGIAP1 fwd | aaaaggatcaaaaATGGCGTACCAATCGGAG | Gibson assembly cloning of AGIAP1 cDNA with AP1 promoter and OCS term into pFP100 |
| AGIAP1 rev | attaaagcaggacTTACACTAACTGGAGAGCGG | |
| OCS\_ AGIAP1 ter\_fwd | ccagttagtgtaaGTCCTGCTTTAATGAGATATGCG | Gibson assembly cloning of OCS ter with AP1 promoter and AGIAP1 CDNA into pFP100 |
| OCS\_terminator\_rev | aaaccaaaatccagtggtacccgggGAGCGGCCGCAGATTTAG | |
